# Bidding for Pauper Practice

**Published:** 1878-03

**Authors:** 


### BIDDING FOR PAUPER PRACTICE.

*To the Editor of the Chicago Medical Journal and Examiner:*

I desire to call the attention of the medical profession, through your Journal, to a practice among physicians in some localities that appears to me to be of doubtful propriety, if not an open violation of the code of ethics. I allude to the custom of physicians entering into competetive bidding for pauper and poor practice at the instance of boards of supervisors. These boards or their committees usually give public notice that bids will be received from physicians for the medical and surgical attendance of the paupers or the poor for a year. And after some such notice as this, the physicians proceed to bid against each other, or each aims to bid lower than his neighbor, and, as a rule, the one that proposes to do the work for the least money is considered the best bidder and gets the contract.

This may be right and in accordance with the code of ethics, but if it is, I do not understand the code.

Whether the custom of bidding for such practice is a common thing all over the country, or is limited to certain portions of this State, is more than I can tell, but one thing I do know, and that is that it is the custom in some localities in the State of Illinois. And I do not now refer to homeopathic or eclectic practitioners, but to the so-called "regular" members of county and district medical societies, and even members of the Illinois State Medical Society.

Can regular physicians engage in competetive bidding for poor practice without violating the letter and spirit of the code? If the advocates of the practice think the code gives such liberty, I would like them to show me where they find it. I understand the code of ethics of the American Medical Association to discountenance all such practices. I understand also that the American Medical Association has declared that all physicians that bid and contract for practice at less rates than those established by a majority of the regular graduates of the same locality, are to be classed as irregular practitioners (Transactions, vol. xx., p. 41). I am aware that the code of ethics is not a law in a legal sense, but it constitutes a code of rules to guide physicians and to promote harmony among them. But in order to be of any value, the code must be observed.

I do not wish to open this question in order to excite a personal controversy, but to have it discussed and settled. I believe there are some who have been bidding for such practice that were not at the time aware that they were violating the code of ethics or any other rules that they were bound to respect; while there are others who claim that they have the right to bid and that the code gives them that right. It is claimed also by some that they bid in order to keep the practice out of the hands of irregulars, but it appears to me that those who bid on that ground place themselves on a level with irregulars if not a little below them, and have to do so in order to get the contract; for, as before remarked, it is the men that will do the work for the least money that are rewarded.

I think that this method is entirely wrong and should be corrected, but if I have taken a wrong view of the question, I stand ready to be corrected and will yield the points whenever convincing arguments are presented by those who differ with me.

Respectfully yours, JOHN WRIGHT, M. D.

Clinton, Ill., Feb. 6th, 1878.

REMARKS :—1. Is it proper to bid for pauper practice? That is a matter of taste.

2. Is it in violation of the code?

This question turns either upon article VII., (Pecuniary acknowledgements) or upon the relation of the physician to the public, and vice versa.

"The physicians of every district shall establish a fee bill, and make it a point of honor to adhere to it." Where is this done? Where are such charges strictly adhered to? Is it not a fact that one physician charges twice and thrice the fee set down as a maximum, while others confine themselves to the minimum rate? The framers of the code themselves, must acknowledge that their stipulation is very good in theory, but a failure in practice.

It was a mistake to have touched the money question in the code, because theoretical rules cannot regulate a matter of such diversity.

The relation between the public and physicians is another point that cannot be decided by written rule, formulated by but one of the two parties; for this is a mutual affair and will regulate itself by mutual understanding. The code requires a physician to stand upon the elevated platform of professional men, full of dignity and wisdom, but little concerned about such a sordid matter as the money which fills the soul of trades people.

This standard is applicable where wealthy persons follow the vocation of physician; and in countries where this is the rule, such a standard is taken by the profession without the dictation of a code of ethics. But in this country, where medical education can be obtained at a comparatively low rate, men without means crowd the benches of the medical colleges and must earn their bread and butter by their practice. They must follow the rules of business men, and no code of ethics can or will hinder them from so doing, because such hindrance would be fatal to their success.

And the public ? Does it regard the physician in any different light than that of a business man ? Does it pay for services an honorarium i. e., a *voluntary* payment tendered in acknowledgement of services ; or does it not rather, either ask the doctor for his bill or wait till such bill is presented, i. e. until it is asked for money ?

As long as the public pays for medical services each practitioner must have his price. Now it is absurd to stipulate that the price for medical services shall be uniform, the same whether the services are rendered by a one year's graduate or by an experienced physician; the same for one whose store of knowledge is represented by " two courses of lectures of twenty weeks each," and for another who has expended time and money in visiting all the large centres of medical science at home and abroad. As practitioners we are business men, and each must know what his services are worth. A beginner, therefore, will naturally bid for pauper practice at a lower rate than a ten years' practitioner if the latter cares at all for such practice.

It was a mistake for the national association to formulate rules regulating the business relation between physicians and the public.

In fact were all physicians refined gentlemen, as they should be, no written code would ever he required ; because the ethical rules for physicians are not and cannot be different from the unwritten laws of propriety and good manners among gentlemen in good society.

So much for one side of the question—that upon which the medical man places himself when proposals are invited from the representatives of the profession.

But there is another side upon which we hasten to place ourselves. It is that which leads us to inquire by what authority or precedent, contracts for medical service are let in the same manner as that which provides for the supplies of provisions, fuel or clothing. If such authority or precedent exist, we should consider it the duty of every medical man resident in such district, to exert his influence as an individual, as a member of a medical organization, and as a tax-paying citizen, with a view to remunerating by a fixed and reasonable sum the individual designated to perform the duties in question. Then let the position be obtainable by competitive examination and the best results will be reached. Not only will the authorities secure the best talent for the sum allowed, but even in an economical view, good can be accomplished, for the extent of the competition annually would be a safe and sure index of the extent to which the salary of such an office could be fairly and safely diminished without doing injustice to the actual necessities of the paupers.—Ed.

---

Mr. Ledley Taylor has devised an arrangement for making visible some of the complex motions of sound waves. A soap bubble solution is made with soap and glycerine, as this makes a more lasting film than water. A hole is cut in a piece of cardboard, and a film of the solution, not too thick to produce bands of color, is made across it. The card is placed on the end of a resonating box which supports a tuning fork. The fork is then thrown into vibrations with a violin bow, and bands of color immediately throw themselves into a pattern with vortex rings of motion and squares and bands of color.

The *Popular Science Monthly* is responsible for the statement that Japanese children thrive on unripe fruit. The absence of children's disorders in the island is attributed to the small amount of cold water used, to the fact that no child is fed artificially, and to the proper attention given to sewerage matters.

In the Massachusetts Charitable Eye and Ear Infirmary, 6575 eye patients, and 2508 ear patients, were treated during 1877. Graefe's operation for cataract was performed on 56 eyes; other cataract operations, 27; iridectomy, 26; enucleation of eye, 36; other operations, 161.

